# Prediction model for anabolic androgenic steroid positivity in forensic autopsy cases – a new tool to the autopsy room

**DOI:** 10.1007/s00414-024-03227-x

**Published:** 2024-04-08

**Authors:** Paula Vauhkonen, Petteri Oura, Pirkko Kriikku, Katarina Lindroos, Mikko Ilari Mäyränpää

**Affiliations:** 1 Forensic Medicine Unit, Finnish Institute for Health and Welfare, Mannerheimintie 166, P.O. Box 30, FI-00271 Helsinki, Finland; 2 Faculty of Medicine, University of Helsinki, Haartmaninkatu 3, P.O. Box 63, FI-00014 Helsinki, Finland; 3 Department of Forensic Medicine, Faculty of Medicine, University of Helsinki, Haartmaninkatu 3, P.O. Box 21, FI-00014 Helsinki, Finland; 4 Forensic Toxicology Unit, Finnish Institute for Health and Welfare, P.O. Box 30, 00271 Helsinki, Finland; 5 Department of Pathology, University of Helsinki, Haartmaninkatu 3, P.O. Box 21, FI-00014 Helsinki, Finland; 6 Helsinki University Hospital, P.O. Box 340, FI-00029 Helsinki, Finland

**Keywords:** Anabolic androgenic steroids, Forensic autopsy, Post-mortem toxicology, Prediction model

## Abstract

Non-prescription use of anabolic androgenic steroids (AAS) is associated with an increased risk of premature death. However, these substances are seldom screened in connection with forensic cause-of-death investigation, unless the forensic pathologist specifically suspects use, often based on a positive AAS use history. Since AAS use is often concealed from others, this practice may lead to mistargeting of these analyses and significant underestimation of the true number of AAS positive cases undergoing forensic autopsy. Thus, more accurate diagnostic tools are needed to identify these cases. The main objective of this study was to determine, whether a multivariable model could predict AAS urine assay positivity in forensic autopsies. We analyzed retrospectively the autopsy reports of all cases that had been screened for AAS during forensic cause-of-death investigation between 2016–2019 at the Finnish Institute for Health and Welfare forensic units (n = 46). Binary logistic regression with penalized maximum likelihood estimation was used to generate a nine-variable model combining circumferential and macroscopic autopsy-derived variables. The multivariable model predicted AAS assay positivity significantly better than a “conventional” model with anamnestic information about AAS use only (area under the receiver operating characteristic curve [AUC] = 0.968 vs. 0.802, *p* = *0.005*). Temporal validation was conducted in an independent sample of AAS screened cases between 2020–2022 (n = 31), where the superiority of the multivariable model was replicated (AUC = 0.856 vs. 0.644, *p* = *0.004*). Based on the model, a calculator predicting AAS assay positivity is released as a decision-aiding tool for forensic pathologists working in the autopsy room.

**Supplementary Information:**

The online version contains supplementary material available at 10.1007/s00414-024-03227-x.

## Introduction

Since the introduction of anabolic androgenic steroids (AAS) to the athlete community, the illicit use of these substances has been a perennial problem in society [1]. Every now and then, non-prescription AAS use is also encountered in the setting of forensic cause-of-death investigation. This is not surprising, since several factors have been identified that may increase the risk of sudden or premature death in connection with supraphysiological dosing these substances. These include pathological cardiac left ventricular and interventricular septum remodeling via hypertrophy and fibrosis [2–5] with subsequent left ventricular dysfunction [6–10]; post-exercise cardiac electrical instability [11]; hypertension [12]; acceleration of coronary plaque formation [9]; and hypercoagulability, predisposing to thrombotic events [13]. Other potentially fatal organ manifestations include acute cholestasis, peliosis hepatis, hepatic tumors [14]; and acute or chronic kidney injury [15]. Some individuals are prone to severe concomitant substance abuse [16–18], and may exhibit aggressive or suicidal behavior, possibly mediated by antisocial personality or other behavioral disorders [19–21]. Indeed, non-therapeutic AAS use seems to be an independent risk factor for premature death [22].

Based on previous forensic autopsy studies, the stereotype of a deceased AAS using person is a young male with overdeveloped musculature, pronounced cardiac hypertrophy, and polydrug use [3, 23–26]. This description correlates with the global epidemiological estimates, indicating that the most typical user group is young to middle-aged males with athletic or personal appearance objectives [27]. On the grounds of these previous publications, Esposito and colleagues recently suggested an investigative protocol for forensic professionals in cases of suspected AAS-induced sudden deaths of young athletes [28]. However, there are multiple other, non-athletic populational subgroups using these substances [29]. Also, AAS use is often concealed from others, including medical professionals [30]. This may result in false negative AAS use anamnesis. The question is, do we see the whole picture?

The decision to request AAS analysis in an autopsy case often rests on the shoulders of an individual forensic pathologist. To ensure the correct targeting of these analyses, more sophisticated diagnostic methods are needed alongside the traditional approach, relying strongly on positive AAS use history. In this study, we aimed to develop further tools to manage this issue by testing, whether a multivariable model could predict the probability of AAS assay positivity in forensic autopsy cases. The model was based on a set of circumferential and macroscopic autopsy-derived variables, consistent with typical baseline data available for the forensic pathologist at the end of the macroscopic autopsy. The performance of the multivariable model was compared to the conventional method with lifetime documentation of AAS use as the only predictor of AAS positivity. The fundamental aim was to convert the multivariable model into a simple calculator to be applied to practical work in the autopsy room.

## Material and methods

### Material

According to Finnish law (the Act (459/1973) and Decree (948/1973) on the inquest into the cause of death), all suspected cases of unnatural death or sudden death without known disease are subjected to medico-legal cause-of-death investigation governed by the police. These include cases where the decedent has not received medical treatment during their last illness, or if death is suspected to be the result of a crime, accident, suicide, poisoning, occupational disease, or medical procedure. In the majority of such cases, forensic autopsy is performed to confirm the cause and manner of death. These autopsies are centralized at the Finnish Institute for Health and Welfare (THL), which has five Forensic Medicine Units located around the country (Helsinki, Turku, Tampere, Kuopio, and Oulu). During the last years, forensic autopsy has been performed in approximately 15 – 16% of all Finnish deaths [31].

#### Inclusion criteria

The study builds on two independent samples: 1) the main sample and 2) a temporal validation sample. The main sample included all cases that were screened for AAS in connection with forensic cause-of-death investigation at any of the THL forensic units, in 2016–2019 (46 subjects, all Caucasian birth-assigned males; 16 AAS screen positive and 30 AAS screen negative). The validation sample was gathered with identical inclusion criteria from cases screened in 2020–2022 (31 subjects, all Caucasian birth-assigned males; 15 AAS screen positive and 16 AAS screen negative).

#### Toxicological analysis

Post-mortem samples were screened for AAS whenever there was background information on use, or if the forensic pathologist had other reasons to suspect use. Post-mortem urine stored in tubes containing potassium fluoride for stability was used for the analysis. During the study period, three laboratories were subcontracted to perform the analyses: the United Medix Laboratories in Helsinki (until January 2019), the laboratory of the National Board of Forensic Medicine in Linköping, Sweden (February 2019–December 2020) and THL Doping Control Laboratory, Finland (January 2021 onwards). In all these laboratories, the analytical procedure was based on chromatographic mass spectrometric identification and confirmation of a large variety of anabolic steroids. The results were reported qualitatively.

#### Circumferential variables

Circumferential variables were derived from the autopsy reports. These included documentation of lifetime AAS use and abuse of other substances (drugs or alcohol), based on available medical records and police investigation at the death scene, the decedent’s house, and police interviews of friends or relatives, where possible.

#### Autopsy derived variables

Macroscopic autopsy findings and organ measurements were obtained from the autopsy reports. The decedent’s weight and height were measured on the autopsy table without clothes. The BMI was calculated using the formula weight (kg) / height squared (m^2^). Description of the body habitus (muscular vs. other, e.g., average build, obese) and evaluation of post-mortem interval (PMI) was documented by the forensic pathologist. Organ weights were measured with calibrated digital scales after transecting the major attachments. Heart width, thickness and length; and right, left and interventricular wall thickness were measured manually using a ruler. Abdominal subcutaneous fat thickness was measured at the umbilicus level after opening the peritoneal cavity.

In case of any injury to an organ (including small lacerations and hematomas) or non-traumatic intraparenchymal bleeding (such as intracerebral hemorrhage), weight/size measurements of the particular organ were excluded from further analysis. None of the cases was documented to have a systemic disease or condition (e.g., sarcoidosis, cancer), systemic inflammation (e.g., sepsis), burns, or advanced putrefaction.

Macroscopic atherosclerotic changes (fatty streaks, plaques and/or calcification) were documented separately for cerebral arteries (circle of Willis), coronary arteries and extra-coronary arteries (aorta and the main branches). Coronary stenosis was defined as narrowing of ≥ 50% of the artery lumen or if deemed clinically significant by the forensic pathologist.

### Statistical analysis

Statistical analysis was performed in R version 4.2.2 (R Foundation for Statistical Computing, Vienna, Austria) and SPSS Statistics version 28.0.0.0 (IBM, Armonk, NY). The threshold for statistical significance was set at *p* = 0.05. Descriptive statistics were given as percentages (%) with frequencies (n) for categorical variables and medians with ranges (minimum—maximum) for continuous variables. Crude comparisons between AAS positive and negative groups were performed using Fisher’s exact test for categorical variables and Mann–Whitney U test for continuous variables. Non-parametric tests were used due to the small sample sizes.

Using the main sample, we constructed a multivariable prediction model for AAS assay positivity. Binary logistic regression was used, with AAS status as the outcome (positive/negative). As conventional maximum likelihood estimates are prone to small sample bias, we performed a penalized maximum likelihood estimation according to the method suggested by Firth [32–34]. The package “logistf” was used in R [35]. Variables showing a statistically significant crude difference between the AAS groups in the main sample were considered for inclusion in the multivariable model. Age, height and weight were a priori included as fixed predictors, as age is associated with AAS use [36], and body size is associated with organ weights [37]. A complete-case approach was used. Multicollinearity was explored by means of the Variance Inflation Factor (VIF) where values < 4 were interpreted as acceptable [38]. Collinear variables were excluded or otherwise modified (e.g., taking the sum of left and right sides). The combination of variables with the highest Cox-Snell R^2^ statistic (R^2^) was taken forward as the final multivariable model [39]. Point estimates of the final model were tabulated with 95% Wald confidence intervals (CI). Finally, a formula for calculating the probability of post-mortem AAS positivity (0.00—1.00) was produced.

In both the main sample and the validation sample, receiver operating characteristic (ROC) curves were used to illustrate the performance of the calculator-based multivariable model relative to a “conventional” model with anamnestic information about AAS use only. Area under the ROC curve (AUC) was the primary statistic for discriminative ability, with higher values indicating better discrimination [40]. DeLong’s test was used to compare the difference in AUC values between the two models. In addition, box plots were used to illustrate the distribution of calculator-based probabilities for AAS positivity in the positive and negative groups; Mann–Whitney U test was used for comparisons.

## Results

### Characteristics of the main sample

The characteristics of the main sample are demonstrated in Table 1.

**Table 1:**
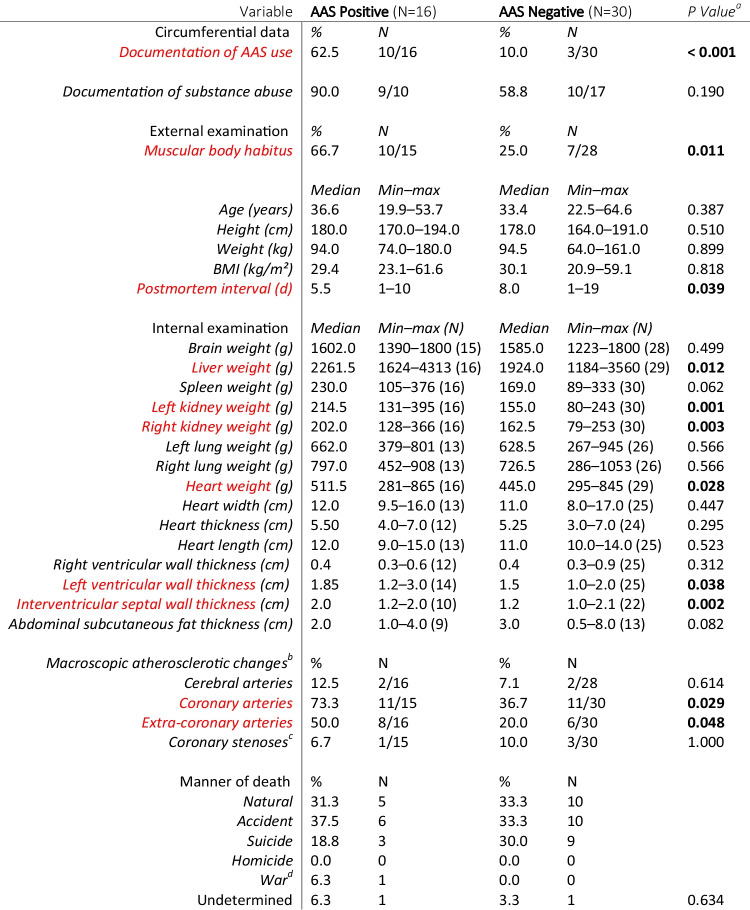
Characteristics of the main sample

Variables showing a statistically significant between-group difference are highlighted

^a^Continuous variables: Mann–Whitney U test; categorical variables: Fisher’s exact test, 2-sided

^b^Fatty streaks, plaques, or calcification

^c^ ≥ 50% luminal narrowing or deemed significant by the forensic pathologist

^d^ Including death caused by the actions of law enforcement

The AAS positive group included a higher percentage of individuals with lifetime documentation of AAS use *(p* < *0.001)*. In most cases, AAS or other hormonal preparations were found in the decedent's apartment. Also, anamnestic abuse of other substances appeared more common among the AAS positive than negative (90.0% vs. 58.8%), but the difference was not statistically significant.

The AAS positive and negative groups were similar regarding age (medians 36.6 vs. 33.4 years), height (180.0 vs. 178.0 cm), weight (94.0 vs. 94.5 kg) and BMI (29.4 vs. 30.1 kg/m^2^). However, the body habitus of an AAS positive individual was significantly more often described as “muscular” (*p* = *0.011*). Subcutaneous fat thickness was slightly lower among the AAS positive than negative, although this difference did not reach statistical significance. The postmortem interval (PMI) ranged from 1 to 19 days, being somewhat longer in the AAS negative group (5.5 vs. 8.0 days, *p* = *0.039*).

The AAS positive had significantly higher liver *(p* = *0.012)*, kidney (*left: p* = *0.001*, *right: p* = *0.003*) and heart weights (*p* = *0.028*); and left ventricular *(p* = *0.038)* and interventricular septal wall thicknesses *(p* = *0.002)*. A higher percentage of the AAS positive had atherosclerotic changes in coronary *(p* = *0.029)* and extra-coronary arteries *(p* = *0.048)*, whereas cerebral artery atherosclerosis and coronary stenoses were infrequent in both groups.

Manner of death was similarly distributed between groups, accidental deaths (trauma or poisoning) forming the largest group altogether.

### Calculator predicting post-mortem AAS assay positivity

In addition to age, height and weight, variables that showed a statistically significant difference between the AAS groups in the main sample were considered for inclusion in the multivariable model. Of these, left and right kidney weights were collinear and were thus summed together. In addition, heart weight, left ventricular and interventricular septal wall thicknesses were collinear and only heart weight was taken forward to the multivariable model.

Table 2 demonstrates the components of the final multivariable model (R^2^ = 0.438). The level of multicollinearity was acceptable (VIFs ≤ 3.4). The multivariable model clearly exceeded the goodness of fit of the “conventional” model, with lifetime documentation of AAS use as the only explanatory variable (R^2^ = 0.306).

**Table 2:** A multivariable model predicting AAS assay positivity among male decedents

Term/variable	Value	Point estimate (95% CI)	Symbol in formula
Intercept	Constant	10.574 (−29.236; 58.779)	-
Age	Continuous, in years	−0.034 (−0.247; 0.098)	*X*_*1*_
Height	Continuous, in centimetres (cm)	−0.094 ( −0.389; 0.116)	*X*_*2*_
Weight	Continuous, in kilograms (kg)	−0.010 ( −0.079; 0.049)	*X*_*3*_
Documentation of AAS use	Yes = 1 No = 0	3.375 (1.033; 7.438)	*X*_*4*_
Muscular body habitus	Yes = 1 No = 0	1.731 ( −0.751; 5.547)	*X*_*5*_
Heart weight	Continuous, in grams (g)	0.005 ( −0.004; 0.016)	*X*_*6*_
Liver weight	Continuous, in grams (g)	−0.002 ( −0.005; 0.001)	*X*_*7*_
Sum of left and right kidney weights	Continuous, in grams (g)	0.017 ( −0.004; 0.052)	*X*_*8*_
Atherosclerotic changes in coronary arteries	Yes = 1 No = 0	1.409 ( −0.896; 4.236)	*X*_*9*_

*AAS* Anabolic androgenic steroids; *CI* Confidence interval

With coefficients for variables *X*_*1*_*—X*_*9*_ taken from Table 2, the probability of AAS positivity (*P*) can be calculated using the following formula:

$$P=1/(1+{e}^{-(10.574-0.034{X}_{1}-0.094{X}_{2}-0.010{X}_{3}+3.375{X}_{4}+1.731{X}_{5}+0.005{X}_{6}-0.002{X}_{7}+0.017{X}_{8}+1.409{X}_{9})})$$

The calculator based on this formula is available in a supplementary file (Calculator predicting post-mortem AAS assay positivity.xlsx).

**Fig. 1:**
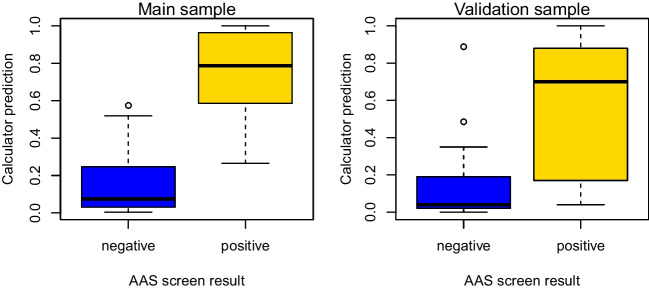
Box plots demonstrating the distribution of calculator scores among the AAS positive and negative groups in the main sample (left) and the validation sample (right). Boxes denote middle quartiles; whiskers denote values within ± 1.5 × interquartile range; and circles denote outliers

**Fig. 2:**
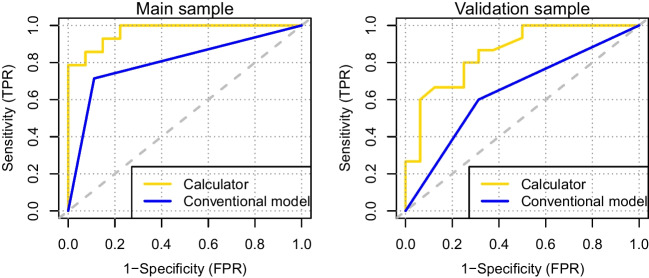
Receiver operating characteristic (ROC) curves demonstrating the discriminative ability of the calculator-based model and the “conventional” model (with lifetime documentation of AAS use as the only explanatory variable) in the main sample (left) and the validation sample (right). TPR = True positive rate, FPR = false positive rate

In the main sample, the distribution of calculator scores showed a statistically significant difference between the AAS positive and negative groups (medians 0.79 vs. 0.07, *p* < *0.001*; Fig. 1). ROC curves based on the calculator and the “conventional” model (with lifetime documentation of AAS use as the only explanatory variable) are presented in Fig. 2. The calculator showed a higher discriminative ability (AUC = 0.968, 95% CI 0.924—1.000) than the conventional model (AUC = 0.802, 95% CI 0.665—0.938), the difference being statistically significant (difference in AUC 0.166, 95% CI 0.051—0.282, *p* = *0.005*).

### Validation of the calculator

The characteristics of the validation sample were highly similar to the main sample (median age 36.9 years, height 180 cm, weight 97 kg, PMI 8 days).

In the validation sample, the distribution of calculator scores showed a statistically significant difference between the AAS positive and negative groups (medians 0.70 vs. 0.04, *p* = *0.001*; Fig. 1). The calculator had a higher discriminative ability (AUC = 0.856, 95% CI 0.725—0.988) than the conventional model (AUC = 0.644, 95% CI 0.470—0.818), the difference being statistically significant (difference in AUC = 0.212, 95% CI 0.068—0.357, *p* = *0.004*; Fig. 2). As such, the superiority of the calculator was demonstrated also in the validation sample.

## Discussion

Previous studies have noted the importance of AAS analysis in connection with forensic cause-of-death investigations, especially in cases of sudden, cardiac death [25, 26, 28]. However, AAS-related deaths are not always due to cardiac causes [23, 24]. Currently, prompt international and national guidelines on AAS testing in various scenarios of cause-of-death investigation are lacking. This may cause inconsistency between individual forensic pathologists, lead to low number of requested analyses and considerable underdiagnosis, and in long term, impair scientific research in this field. For example, altogether 33 643 forensic autopsies were performed in Finland between 2016 and 2019 [31], but AAS assay was conducted in only 46 cases (0.1%). Of these, 34.8% turned out to be positive. Considering the global and national estimates on AAS use prevalence in modern society [27, 29, 41], the actual number of Finns using these hormones and undergoing forensic autopsy is most likely significantly higher.

The present study was designed to determine, whether a multivariable regression model could predict AAS assay positivity in forensic autopsy cases. The second objective was to evaluate the predictive value of the multivariable model, by comparing it to the conventional model with lifetime AAS use documentation as the sole predictor of AAS positivity. The key variables were chosen from a set of circumferential, external and internal examination data, available to the forensic pathologist at the end of the macroscopic autopsy – before making the decision to proceed with further toxicological analysis. We found that a nine-variable model predicted AAS positivity markedly better than documentation of lifetime AAS use alone. The calculator based on the multivariable model showed excellent discriminative ability in both the main sample and the independent validation sample and exceeded the AUC scores of the conventional model.

In the main sample, crude between-group differences were observed in lifetime AAS use documentation; description of the body habitus; PMI; weights of the heart, liver and kidneys; left ventricular and interventricular septum thickness; and the frequency of coronary and extra-coronary atherosclerotic changes. In both groups, the median weights of the organs listed above were at or above the upper quartile of the normal distribution in general adult population [37]. To some extent, this may be explained by the observed high BMI of the sample [37, 42]. However, high BMI itself may reflect obesity and abnormal metabolic state, in which case the organomegaly may not be purely physiological. In the case of AAS use, the high BMI seems to reflect overt muscularity, and the extent to which traditional organ weight reference tables can be used for comparison, remains obscure.

Nevertheless, the high median heart weight observed in the AAS positive group clearly indicates pathologic adaptation [43]. Our findings support the previously noticed associations with AAS use and cardiac hypertrophy, and abnormal left ventricular and interventricular septal wall measurements [2–4]. Advanced subclinical coronary atherosclerosis has also been described before, with cumulative duration of AAS use increasing the plaque volume [9]. Young AAS using males have been shown to have disturbed shear rate in brachial artery along with heightened high-sensitivity C-reactive protein (hs-CRP) [44], which has been reported to associate with increased susceptibility to coronary and extra-coronary atherosclerosis [45]. However, comparative autopsy studies regarding the severity of atherosclerosis in different AAS using age groups are lacking.

Higher liver and kidney weight among the AAS positive warrants further investigations. This finding could be related to congestion, considering the rich vascular supply of these organs. Indeed, visceral congestion has been shown to increase renal weight, yet liver weight seems to be affected only by blood loss [42]. Congestion of these organs has previously been reported in case studies of deceased AAS using individuals, most likely reflecting the terminal stages associated with the cause and manner of death [3, 46]. However, a case report by Luke et al. described actual renal hypertrophy in a deceased AAS using weightlifter [47]. Experimental data suggests that several mechanisms may trigger renal growth, and this hypertrophy may be either physiological or pathological, as in diabetic nephropathy [48]. Some of the maladaptive pathways are stimulated by testosterone [15], the end of the route being fibrosis and renal atrophy. It should also be recognized that organ hypertrophy in connection with AAS use may be caused by other concomitantly used drugs, such as insulin-like growth factor (IGF) or growth hormone (GH) [15, 49, 50]. Due to their structural similarity to endogenous hormones, these drugs are not readily available in post-mortem toxicological screens.

The observed PMI medians in the main and validation samples were close to the average PMI of all Finnish forensic autopsies during the study period (8.5 days; personal communication with Aki Eklin, Senior Planning Officer at the Finnish Institute for Health and Welfare), which is mainly due to prolonged cold storage of the cadavers before the autopsy. The difference in PMI between the AAS positive and negative groups in the main sample is unlikely related to AAS use per se. One must consider the possibility of post-mortem drug degradation due to bacterial activity, which could cause true AAS positive cases to turn negative as the PMI increases. For this reason, PMI was initially considered as one explanatory variable in the multivariable model, but there was no additional benefit for model performance. This does not necessarily mean the issue is irrelevant, but a further evaluation of this perspective would require a larger study sample. Meanwhile, there is no reason to refrain from urinary AAS analysis based solely on prolonged PMI, since exogenous anabolic steroids and their metabolites are often found in forensic post-mortem urine samples in large quantities [51], and their overall stability in biological fluid samples is good [52].

In the validation sample, the calculator scores were mainly below 0.4 for the AAS negative, while there was more dispersion among the AAS positive. Some of this dispersion may be explained by the duration of AAS use and its timing related to death, since AAS are often used in cycles with breaks of variable length in between. It seems unlikely that a single cycle of AAS use would result in significant atherosclerosis or permanent change in organ size; yet it would likely be detected in the urine screen, if timed within a few weeks or a couple of months prior to death [53]. Some of the observed dispersion may also be explained by concomitant drug use or somatic disease. Further work and larger samples are thus needed to calibrate the calculator and to determine its applicability in cases with congenital and systemic disease.

Taking these limitations regarding implementation into account, the calculator still clearly exceeds the value of lifetime AAS use documentation when deciding on possible AAS analysis and can be recommended as an additional tool to the autopsy room. It is potentially beneficial in all forensic cases with no known somatic etiology for organomegaly, and especially in cases with obscure or lacking lifetime AAS use anamnesis. It may be also helpful in determining whether testing for AAS or for genetic disease would be relevant in cases with cardiac hypertrophy – the latter also concerning the next of kin. A high calculator score should prompt testing while the significance of a lower score is to be proportioned to the circumstances and possible juridical aspects of each death. In cases with high calculator score but negative urine assay, additional body hair analysis should be considered if available, as it may offer longer detection time (up to several months) than urine for previous AAS use [54].

We recognize the limitations of our study. First, this was a retrospective study and several forensic pathologists have performed the autopsies. The Finnish forensic autopsy reports are semi-structured and allow freedom of choice regarding certain practical matters. For example, the hearts may be weighed as a whole, after washing out all blood and blood clots; or after transecting the organ. Similarly, the liver may be weighed with or without gallbladder. As a result, there may be minor differences in organ measurements between operating pathologists, but this error is unlikely systematic in the study sample. Also, due to the retrospective nature of the study, only systematically documented organ measurements were available as potential variables in the model. These did not include all possibly relevant measurements, such as testicular size, which may decrease during active AAS use [55]. Second, qualitative variables of subjective origin were included in the predictors. Naturally, classifying the body habitus as “muscular” without objective measurements of the body composition, is prone to bias. Both the AAS positive and negative group demonstrated above normal median BMI, and despite the trend of the AAS positive having less abdominal subcutaneous fat, there were no statistically significant differences between the groups in either of these variables. Yet, the AAS positive individuals were significantly more often described as having a muscular body habitus vs. something else. This indicates that subtle differences between normal and abnormal muscularity have been observed by the forensic pathologists. Furthermore, there are currently no other easily implementable, quantitative measurements available in the autopsy room to replace this variable. Third, the study sample was rather small. This carries the risk of model overfitting. To reduce this risk, a penalized regression method was chosen. Last, the study sample included only males, so the results may not be applicable to females with AAS use. It should also be emphasized that our model is based on national samples, which may reduce the applicability of the calculator to other forensic autopsy environments.

## Conclusions

This study introduced a prediction model for AAS urine assay positivity in forensic autopsy cases. The multivariable model performance was excellent in both the main and the validation sample. We believe that introducing the model based calculator to the autopsy room will increase knowledge on the issue and hopefully, encourage AAS testing especially in cases with obscure lifetime AAS use history. The calculator could also serve as a basis for developing a diagnostic tool for living patients suspected to have used AAS, as new imaging methods enabling more accurate organ size estimation emerge.

### Supplementary Information

Below is the link to the electronic supplementary material.

Supplementary file1 (XLSX 13 kb)

## Data Availability

The datasets generated during the current study are available from the corresponding author on reasonable request.
